# Terpinen-4-ol triggers autophagy activation and metacaspase-dependent apoptosis against *Botrytis cinerea*

**DOI:** 10.3389/fmicb.2025.1600831

**Published:** 2025-08-29

**Authors:** Kunchun Wang, Zhenbo Li, Shengnan Shen, Lei Wang, Huizheng Wang, Delong Li

**Affiliations:** ^1^Agricultural Technology Service Center of Linzi District, Zibo, China; ^2^Shandong Agricultural Technology Extension Service Center, Jinan, China; ^3^College of Plant Health and Medicine, Engineering Research Center for Precision Pest Management for Fruits and Vegetables of Qingdao, Qingdao Agricultural University, Qingdao, China; ^4^Zibo Linzi District Municipal Gardening and Sanitation Service Centre, Zibo, China; ^5^College of Agricultural Engineering and Food Science, Shandong University of Technology, Zibo, China

**Keywords:** antifungal activity, autophagy, chemical control, apoptosis, metacaspase-dependent, gray mold

## Abstract

*Botrytis cinerea*, a necrotrophic phytopathogen responsible for gray mold disease, poses a significant threat to global postharvest horticultural production due to substantial spoilage of fruits and vegetables. This study systematically investigated the antifungal efficacy and molecular mechanisms of terpinen-4-ol against *B. cinerea.* Terpinen-4-ol exhibited a broad-spectrum of antifungal activity, significantly inhibiting both mycelium growth and conidial viability of *B. cinerea*. Further analyses revealed that terpinen-4-ol disrupted cell membrane integrity and induced reactive oxygen species (ROS) accumulation. The inhibitory effect may be attributed to its ability to promote ROS accumulation and induce autophagy activity, thereby disrupting the intracellular redox balance and autophagic processes in fungi, ultimately leading to apoptosis via a metacaspase-dependent pathway. Altogether, these findings revealed a specific antifungal mechanism of terpinen-4-ol against *B. cinerea*, suggesting its potential as an effective preservative for postharvest preservation of fruits.

## Introduction

Gray mold, caused by *Botrytis cinerea*, is widely acknowledged as the most economically important postharvest disease impacting the global production of food and ornamental plants (Williamson et al., 2007). Traditionally, chemical control through the application of fungicides has been the primary method for managing gray mold (Smilanick et al., 2010). However, the growing global concerns regarding the environmental impacts and human health risks associated with chemical residues have spurred interest in developing sustainable alternatives (Combrinck et al., 2011; Hussin et al., 2021). Among these alternatives, plant-derived essential oils (EO), have emerged as promising candidates due to their broad-spectrum antimicrobial activity, biodegradability, and eco-friendly properties (Pan et al., 2023; Utama et al., 2020), demonstrating significant potential in controlling plant pathogens and extending the shelf-life of perishable commodities (Doyle and Stephens, 2019).

Numerous studies have demonstrated the *in vitro* efficacy of EO in inhibiting postharvest fungi (Lopez-Reyes et al., 2013). Among these, tea tree oil (TTO) extracted from *Melaleuca alternifolia* has been widely used to treat various conditions in human and animal, and is considered as an effective alternative to the most commonly used antifungal agents (Shao et al., 2013; Terzi et al., 2007). In *in vitro* experiments showed, TTO vapour effectively suppresses conidial germination and mycelial growth of the main postharvest pathogens including *Fusarium* spp. and *Rhizopus stolonifera* (Jing et al., 2014; Jung et al., 2014). However, further investigation is required to explore the volatile active constituents of TTO and their antifungal activity against phytopathogens.

Terpinen-4-ol [3-cyclohexen-1-ol,4-methyl-1-(1-methylethyl)-, (R)-] is a terpene that serves as the primary component of TTO and is also found in various other plants, such as *Alpinia zerumbet* and *Eucalyptus* species from Hajeb Layoun arboreta in Tunisia (De et al., 2018; Hart et al., 2000; Swords and Hunter, 1978). Additionally, terpinen-4-ol has been shown efficacy against fungal species such as *Aspergillus flavus*, *Candida* spp., *Saccharomyces cerevisiae*, and other yeast species, primarily through membrane-targeted mechanisms (Avis and Belanger, 2001; Yalage Don et al., 2021), including increasing cell membrane permeability, compromising cell membrane integrity, inducing ROS accumulation, affecting protein and DNA synthesis, and reducing ATP content (Ren et al., 2024; Yu et al., 2015; Zhang et al., 2018). Additionally, it has also been shown to improve disease resistance in strawberry fruit by activating the phenylpropanoid metabolism pathway (Li et al., 2020). Given its antimicrobial properties, terpinen-4-ol has garnered significant scientific interest (Nogueira et al., 2014).

Based on this, our study systematically evaluates the antimicrobial potential of terpinen-4-ol against *B. cinerea*, and the possible mechanism. The study revealed that terpinen-4-ol exhibits broad-spectrum antifungal activity, exerting inhibitory effects against both fungi and oomycetes. The mechanistic investigations indicate that terpinen-4-ol disrupts plasma membrane integrity, induces ROS accumulation, triggers ER-phagy and autophagy processes, and activates metacaspase-dependent apoptosis in *B. cinerea*. Moreover, its effectiveness in reducing pathogenicity on tomato leaves, tomatoes, and strawberries underscores its applicability in postharvest disease management.

## Materials and methods

### Fungal strains

The *B. cinerea* strain 05.10 was maintained in our laboratory. Other phytopathogenic strains, including *F. oxysporum*, *F. graminearum*, *Valsa mali*, *Phomopsis vaccinii*, *Pestalotiopsis theae*, *Rhizoctonia solani*, and the oomycete *Phytophthora capsici*, were also cultured on potato dextrose agar (PDA) at 25°C under dark conditions.

The BcRtn1-GFP, BcIlv2-GFP, BcGFP-SKL, and GFP-BcAtg8 strains were described previously (Wang et al., 2023) and available from the corresponding author’s laboratory. The *ΔBcMca1*, *ΔBcMca2*, and *ΔBcMca1Mca2* mutants (Wang et al., 2023) were maintained in our laboratory. All these strains were also cultured on potato dextrose agar (PDA) at 25°C under dark conditions.

### Materials and reagents

Fresh tomato leaves were harvested from greenhouse-grown plants, while fresh tomatoes and strawberries were obtained from local markets. Terpinen-4-ol (95% purity; CAS: 20126-76-5) was purchased from Macklin (Shanghai, China). Propidium iodide (PI), 2′,7′-dichlorofluorescein diacetate (DCFH-DA), N-(3-triethylammoniumpropyl)-4-(6-(4-(diethylamino) phenyl) hexatrienyl) pyridinium dibromide (FM4-64) and annexin V-PE were purchased from Beyotime Biotechnology (Shanghai, China).

### Antifungal activity of terpinen-4-ol on plant pathognes *in vitro*

Terpinen-4-ol was added to PDA to achieve the desired final concentration of 0, 0.2, 0.4 and 0.8 μl/ml. Mycelial plugs (5 mm in diameter) of the plant pathogens and the *B. cinerea* mutants Δ*BcMca1*, Δ*BcMca2*, and Δ*BcMca1Mca2* were inoculated onto PDA plates, which were then incubated at 25°C in the dark for 3 days. The colony diameter was measured, excluding the original plug size. Conidial germination assays were conducted in a 96-well microtiter plate, with 180 μl of spore suspension (4.6 × 10^4^ spores/ml) in each well. The conidial suspensions were treated with different concentrations of terpinen-4-ol (0, 0.2, 0.4 and 0.8 μl/ml). The germination rate of the conidia was then estimated after incubation at 25°C for 6 h. Three independent technical replicates were performed.

### Testing the inhibitory effect of terpinen-4-ol on pathogenesis

Conidia of B05.10 were collected from 7-day-old PDA cultures. Conidial concentration was determined microscopically using a hemocytometer and adjusted to 4.5 × 10^4^ conidia/ml. Detached leaves from 4-week-old tomato plants received 30 μl terpinen-4-ol sprays (0, 0.2, 0.4 or 0.8 μl/ml). After 4 h air-drying, leaves were inoculated with mycelial plugs. The antifungal activity was further assessed using commercially available mature tomatoes and strawberry fruits with artificial equatorial wounds (diameter 1 mm). The wounds were sprayed with 0.4 μl/ml terpinen-4-ol or H_2_O for 4 h, then inoculated with either 5-mm mycelial plugs or 20 μl of conidial suspension, and finally incubated in an airtight box. After 3 days, fruits were maintained at room temperature (95% humidity) for a further 3 days before the lesion diameters were measured.

### Fluorescence microscopy

The conidia suspension of *B. cinerea* was inoculated into 100 ml of yeast extract-peptone-dextrose (YEPD) liquid medium and incubated at 25°C at 120 rpm for 24 h. The *B. cinerea* mycelia were treated with 0.1 μl/ml of terpinen-4-ol, while a control group remained untreated. Following an additional 4 h of incubation under the same conditions, the mycelia were harvested and stained with PI (20 μg/ml) to assess cell membrane integrity and with DCFH-DA (10 μM) to detect intracellular ROS. Apoptosis was determined by Annexin V-PE assay. Fluorescence was examined using an Olympus fluorescence microscope (Tokyo, Japan). All experiments were performed according to the protocol described in the kit instructions.

To examine whether terpinen-4-ol affects selective or non-selective autophagy, conidia of these strains expressing GFP-tagged markers (BcRtn1-GFP, BcIlv2-GFP, BcGFP-SKL, and GFP-BcAtg8) were cultured in YEPD liquid medium at 25°C for 24 h. The mycelia were then exposed to 0.1 μl/ml of terpinen-4-ol or H_2_O for 4 h, as previously described. The samples were stained with FM 4–64, and the fluorescence was examined using an Olympus fluorescence microscope (Tokyo, Japan) (Meng et al., 2025).

### Protein extraction and Western blotting

The wild-type strain B05.10 and gene-overexpressing strains, including the GFP-BcAtg8, BcRtn1-GFP, BcGFP-SKL, and BcIlv2-GFP strains, were cultivated in YEPD liquid medium at 25°C in a 120-rpm shaker for 24 h. Subsequently, the cultures were treated with terpinen-4-ol or H_2_O for an additional 4 h as previously described. Mycelia were then harvested and resuspended in protein extraction buffer. Equal volumes of protein extracts from each strain were separated by SDS-PAGE and transferred to polyvinylidene fluoride membranes. Immunoblotting was performed using an anti-GFP antibody (Cat# 32146, Thermo Fisher Scientific) at a dilution of 1:5,000, with an anti-actin antibody (Abcam, Cambridge, MA, USA) serving as a reference.

### RNA preparation and quantitative real-time PCR (qRT-PCR) analysis

For the analysis of *BcMac1* and *BcMac2* gene expression, total RNA was extracted from *B. cinerea* mycelia under two conditions: *B. cinerea* treated with either terpinen-4-ol or H_2_O, and *B. cinerea* treated with either terpinen-4-ol or H_2_O during subsequent pathogenicity assays. RNA isolation was carried out using the TRIzol method (TaKaRa, Japan) in accordance with the manufacturer’s instructions. The PrimeScript RT Reagent Kit with gDNA Eraser (TaKaRa) was employed for reverse transcription of total RNA. TB Green® Premix Ex Taq (TaKaRa) was used to qRT-PCR analyses. Transcript levels were normalized to the expression of the β-actin gene.

### Statistical analyses

All experimental data are presented as the means ± the standard errors. Statistical differences were analyzed using analysis of variance (ANOVA) and followed by Duncan’s multiple range tests in SPSS 21.0 (SPSS Inc.). A value of *p* < 0.05 was considered statistically significant.

## Results

### Antifungal activity of terpinen-4-ol against plant pathogens

**Figure 1:**
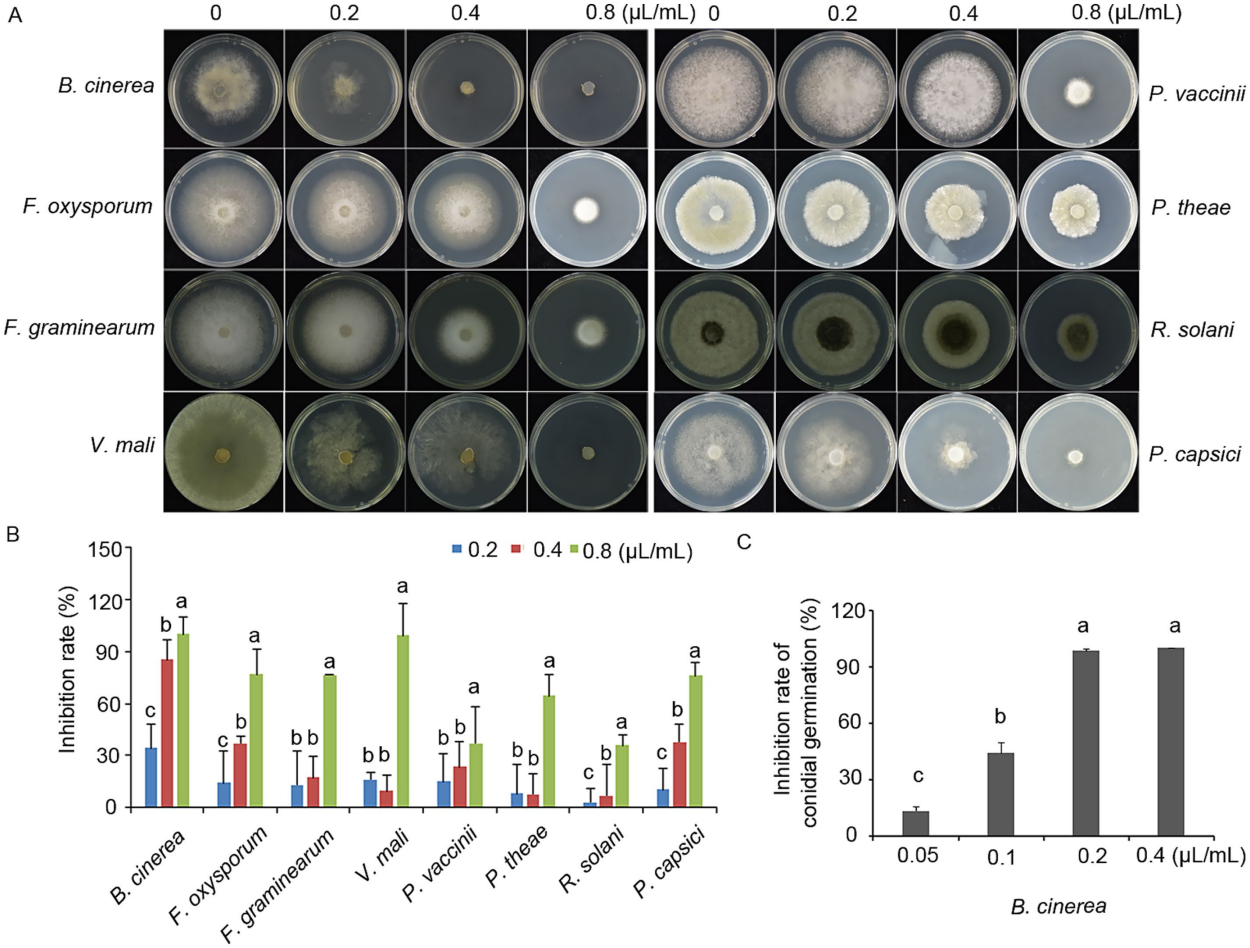
Inhibitory effects of terpinen-4-ol on mycelial growth. **(A)** terpinen-4-ol inhibits the mycelial expansion of phytopathogens after 4 days of growth on PDA plates supplemented with increasing concentrations of terpinen-4-ol. **(B)** Statistical analysis of inhibition rate. **(C)** Inhibition rate of conidial germination in *Botrytis cinerea* under different concentrations of terpinen-4-ol. Each value represents the mean of triplicate measurements, while the vertical bar indicates the standard error. Different letters denote statistically significant differences (*p* < 0.05).

The antifungal efficacy of terpinen-4-ol was evaluated against eight plant pathogens, including fungal and oomycete species, by measuring colony diameter on PDA. Terpinen-4-ol exhibited significant antifungal activity against all eight pathogens, with colony growth inhibited in a concentration-dependent manner (Figure 1A). In the control group, the colony exhibited unrestricted radial expansion, whereas terpinen-4-ol-treated colonies displayed concentration-dependent growth retardation with significantly reduced final diameters. Notably, complete mycelial growth inhibition of *B. cinerea* and *V. mali* was achieved at 0.8 μl/ml terpinen-4-ol. From the perspective of antifungal activity, terpinen-4-ol exhibited the strongest inhibitory effect against *B. cinerea*, with an inhibition rate of 86% at a concentration of 0.4 μl/ml (Figure 1B). Consequently, *B. cinerea* was selected for further mechanistic studies due to its exceptional sensitivity.

To assess the inhibitory effect on conidial germination, *B. cinerea* conidial suspensions were prepared and incubated on slide containing terpinen-4-ol at concentrations of 0, 0.05, 0.1, 0.2, 0.4 μl/ml. Conidial germination rates were suppressed by terpinen-4-ol in a concentration-dependent manner, showing significant inhibition at 0.2 μl/ml (Figure 1C). These results demonstrate that terpinen-4-ol possessed an ability to impair both mycelial growth and conidial germination in *B. cinerea*.

### Terpinen-4-ol disrupts plasma membrane integrity and induced ROS accumulation

**Figure 2:**
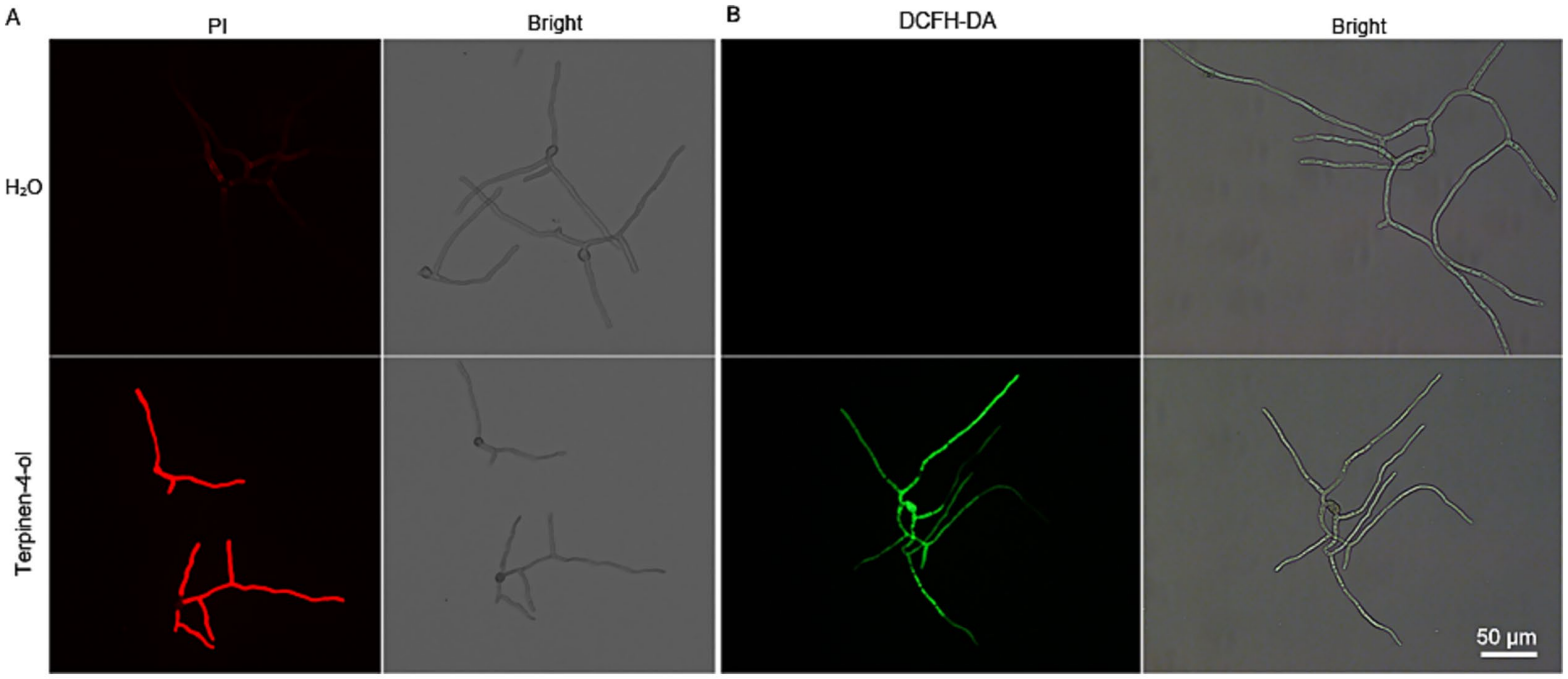
Effects of terpinen-4-ol on cell membrane integrity ROS accumulation. **(A)** Cell membrane integrity after conidial germination treated with terpinen-4-ol was observed using fluorescent dye PI. **(B)** ROS accumulation in conidial germination exposed to terpinen-4-ol for 4 h at 25°C, were visualized by fluorescent staining with DCFH-DA.

To further investigate the effect of terpinen-4-ol on *B. cinerea* plasma membrane integrity, the cell membrane integrity was assessed by PI staining. Compared to the control group, terpinen-4-ol-treated mycelia exhibited pronounced red fluorescence (Figure 2A). This result demonstrates that terpinen-4-ol has the capacity to disrupt cell membrane integrity. Additionally, many plant derived compounds strongly induced ROS production, therefore ROS accumulation was monitored using 2′,7′-dichlorodihydrofluorescein diacetate (DCFH-DA) staining. The terpinen-4-ol-treated group showed a significant increase in fluorescence intensity, whereas no such increase was observed in the control group (Figure 2B). These findings suggest that terpinen-4-ol exerts antifungal activity against *B. cinerea* by disrupting plasma membrane integrity and promoting ROS accumulation.

### Terpinen-4-ol induces ER-phagy and autophagy

**Figure 3:**
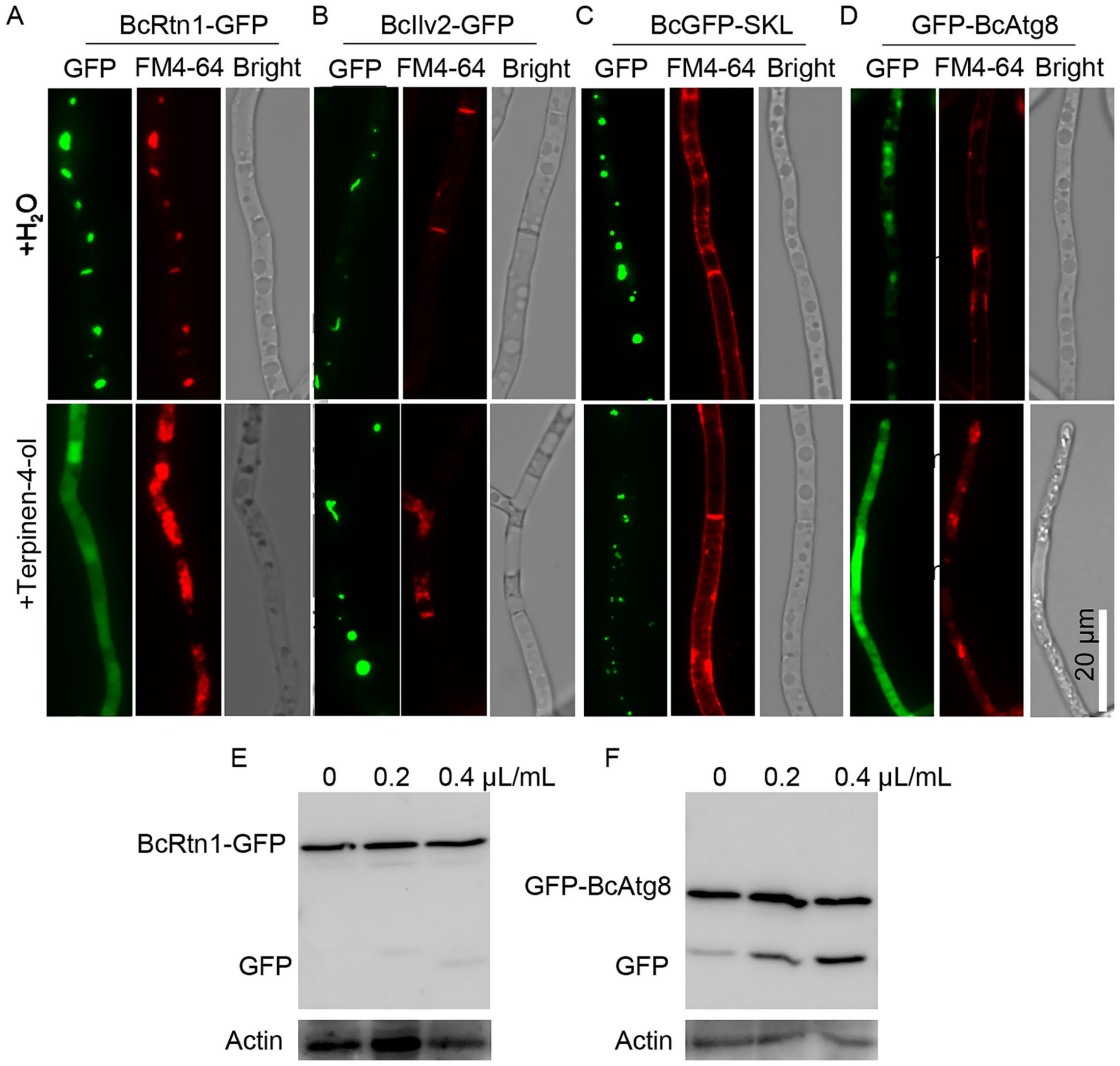
Effects of terpinen-4-ol on *Botrytis cinerea* autophagy. Strains BcGFP-SKL **(A)**, BcRtn1-GFP **(B)**, BcIlv2-GFP **(C)**, and GFP-BcAtg8 **(D)** were incubated in YEPD for 12 h and treated with terpinen-4-ol for 4 h. Then fluorescence was observed with a microscope after staining with FM4-64 for 30 to 45 min. Immunoblot analysis of BcRtn1-GFP **(E)** and GFP-BcAtg8 **(F)** proteolysis.

To investigate whether terpinen-4-ol induces ER-phagy in *B. cinerea*, strains expressing fluorescent markers were analyzed. In the BcRtn1-GFP (ER marker) strain treated with terpinen-4-ol, GFP fluorescence was localized in the cytoplasm and vacuole, whereas GFP fluorescence in the BcRtn1-GFP strain without terpinen-4-ol treatment mainly localized in the ER (Figure 3A). In contrast, mitochondria-localized *BcIlv2-GFP* and peroxisome-targeted *BcGFP-SKL* strains treated with terpinen-4-ol showed no difference from control group (Figures 3B,C). Furthermore, the autophagic flux was analyzed using GFP-BcAtg8. GFP fluorescence was detected in both the cytoplasm and vacuoles of the GFP-BcAtg8 strain treated with terpinen-4-ol, confirming the induction of autophagy (Figure 3D). Next, the process of autophagy was observed through the use of immunoblotting. The results showed that the proportion of free GFP in the terpinen-4-ol-treated mycelia was significantly higher than that of the H_2_O-treated mycelia (Figures 3E,F). Collectively, these results indicated that terpinen-4-ol specifically triggered ER-phagy and autophagy in *B. cinerea*, but did not affect mitophagy and peroxisomal degradation.

### Terpinen-4-ol induces apoptosis dependent on metacaspases

**Figure 4:**
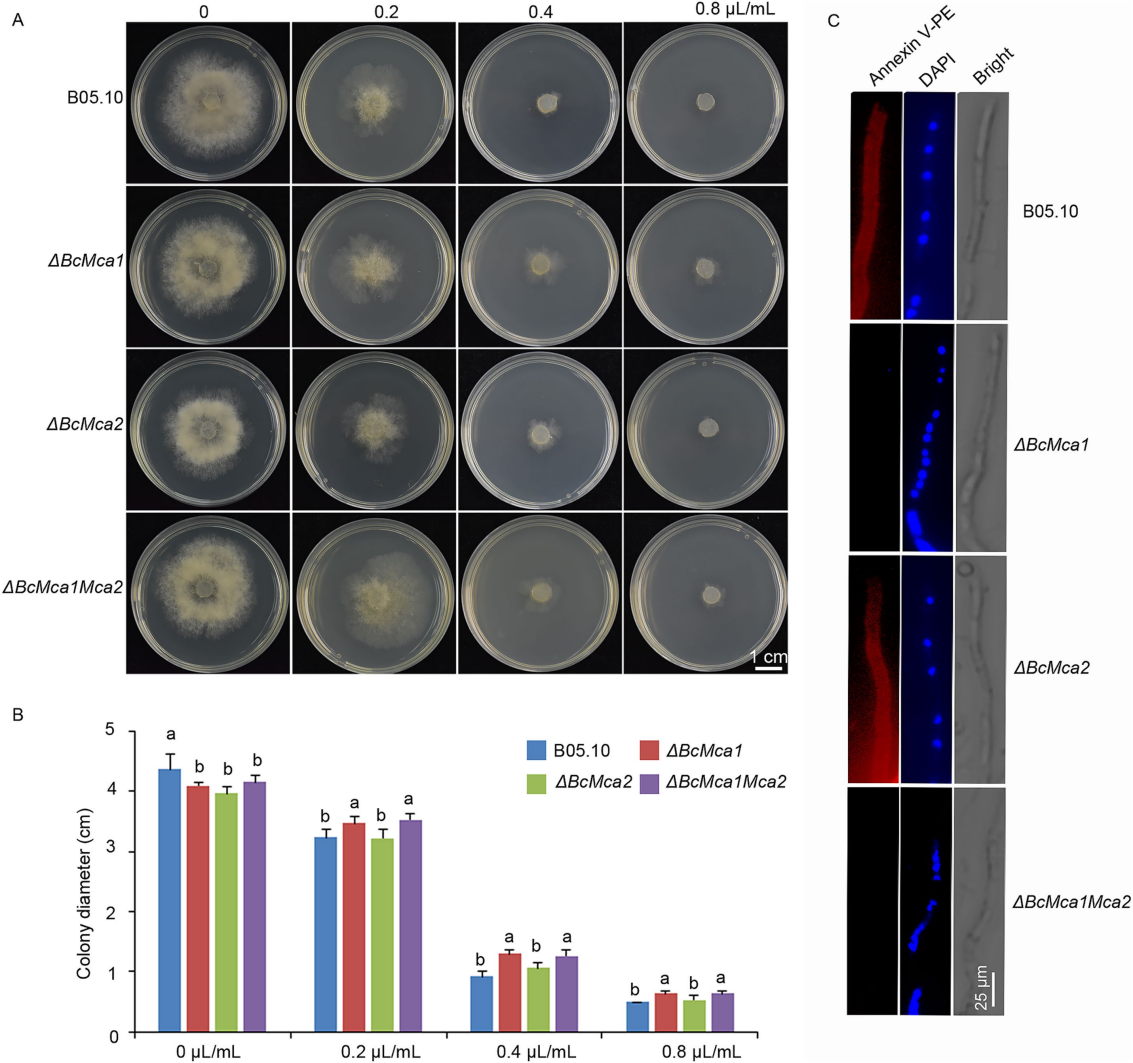
Effects of terpinen-4-ol on metacaspases mutant in *Botrytis cinerea*. **(A)** *ΔBcMca1* and *ΔBcMca1Mca2* strains exhibited resistance to terpinen-4-ol, *ΔBcMca2* strain show no resistance on PDA medium after 3 days under terpinen-4-ol stress. **(B)** Inhibition rate in three mutants. **(C)** Detection of apoptosis in B05.10 and mutants using Annexin V-PE and DAPI staining. Each value represents the mean of triplicate measurements, while the vertical bar indicates the standard error. Different letters denote statistically significant differences (*p* < 0.05).

Previous studies have shown that terpinen-4-ol induces ROS accumulation, a known trigger of apoptotic cell death. In yeast, the metacaspase Yca1 mediates oxidative stress induced programmed cell death. To investigate the role of metacaspases in terpinen-4-ol-induced apoptosis in *B. cinerea*, we analyzed the expression levels of *BcMca1* and *BcMca2* in B05.10 mycelia treated with either terpinen-4-ol or H_2_O. Notably, terpinen-4-ol significantly upregulated *BcMca1* expression (Supplementary Figure S1). To further confirm this observation, we investigated two single-gene deletion mutants (Δ*BcMca1* and Δ*BcMca2*) as well as a double-deletion mutant (Δ*BcMca1Mca2*) strains in our further study. The antifungal sensitivity of these mutants was assessed by culturing the wild-type B05.10 strain and mutants on PDA amended with gradient concentrations of terpinen-4-ol. After 4 days, the Δ*BcMca1* and Δ*BcMca1Mca2* mutants showed decreased sensitivity to terpinen-4-ol compared to that of B05.10. However, Δ*BcMca2* displayed no phenotypic divergence from B05.10 (Figures 4A,B). To further evaluate apoptosis, we conducted annexin V-PE and DAPI staining. Notably, POH treatment induced phosphatidylserine exposure on the outer membrane leaflet in both B05.10 and *ΔBcMca2* mutants (Figure 4C); whereas no such exposure was detected in either *ΔBcMca1* or *ΔBcMca1Mca2* mutants. Based on the above results we speculated that terpinen-4-ol activates apoptosis in *B. cinerea* through the metacaspase BcMca1-dependent pathway.

### Antifungal efficacy on pathogenicity

**Figure 5:**
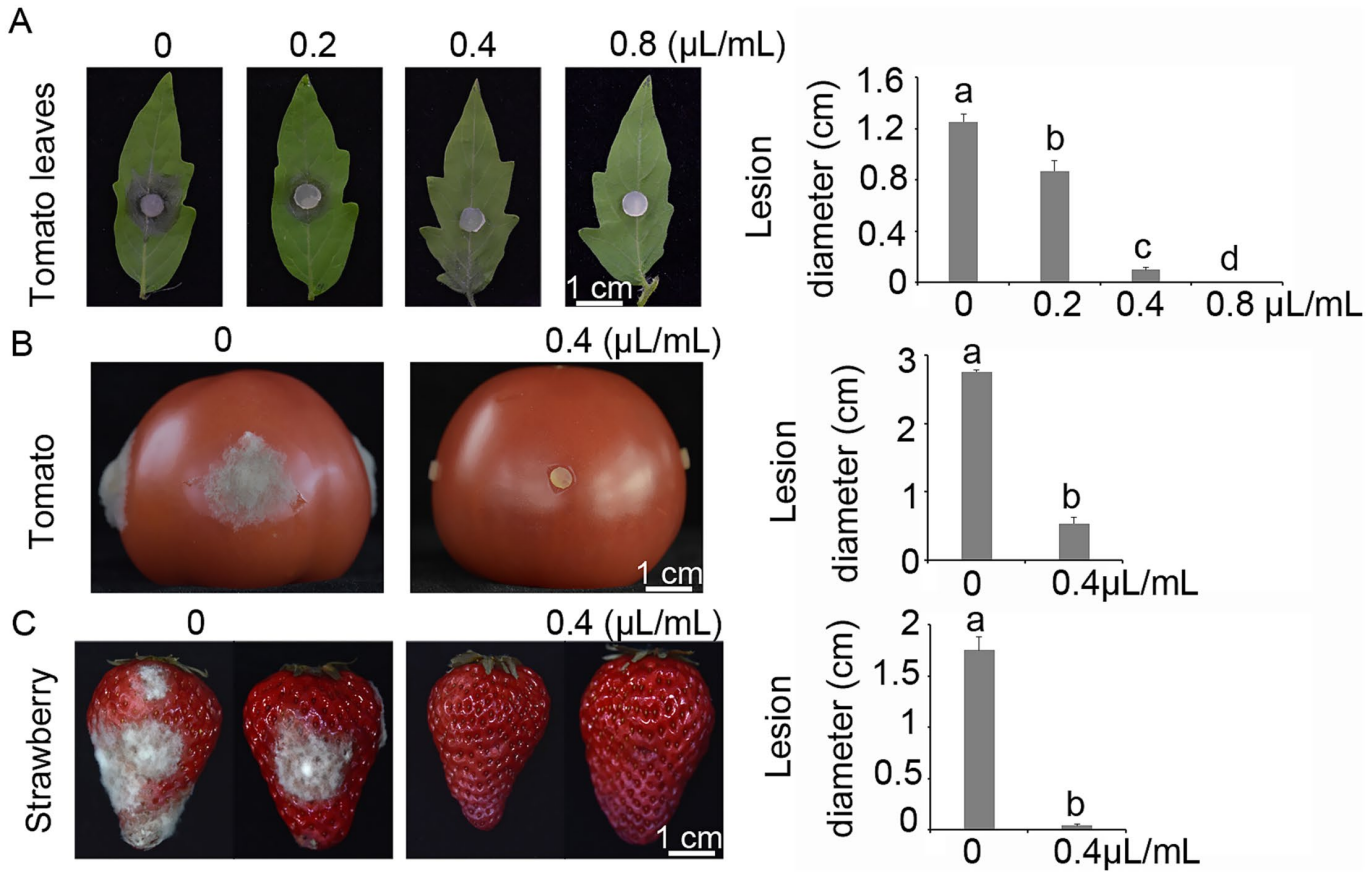
Terpinen-4-ol impairs the pathogenicity of *Botrytis cinerea* on tomato plants and fruits of tomato, grapes, and strawberry. **(A)** Tomato leaves treated with or without terpinen-4-ol were inoculated with mycelial plugs with or without terpinen-4-ol, and incubated in a humid chamber at 25°C. **(B)** Tomatoes treated with or without terpinen-4-ol were inoculated with mycelial plugs, and incubated in a humid chamber at 25°C. **(C)** Strawberry were inoculated with 10 μl droplets of conidial suspension with or without 0.4 μl/ml terpinen-4-ol, and incubated in a humid chamber at 25°C.

The potential inhibitory effect of terpinen-4-ol on the pathogenicity of *B. cinerea* was evaluated using detached tomato leaves, tomato and strawberry fruits. In the detached leaf assay, terpinen-4-ol significantly reduced the pathogenicity of *B. cinerea* in a dose-dependent manner, with a notable reduction in lesion diameter observed at 2 days post-inoculation (dpi) (Figure 5A). Additionally, the antifungal efficacy of terpinen-4-ol was further evaluated on tomato fruits. After 3 days of storage, mycelial plugs of *B. cinerea* inoculated onto tomato treated with terpinen-4-ol (0.4 μl/ml) exhibited significantly smaller lesion diameters compared to those untreated controls (Figure 5B). Similarly, conidial suspensions of *B. cinerea* inoculated onto strawberries treated with terpinen-4-ol (0.4 μl/ml) resulted in significantly reduced lesion diameters after 3 days of storage (Figure 5C). These results demonstrated that terpinen-4-ol effectively inhibited the pathogenicity of *B. cinerea* on both tomato and strawberry fruits. We further examined the expression level of the *BcMac1* and *BcMac2* genes in tomato leaves following terpinen-4-ol treatment during the pathogenicity assay. The results revealed that *BcMac1* expression was significantly higher than that of *BcMac2*, indicating that terpinen-4-ol treatment induced the apoptosis in gray mold during host infection.

## Discussion

Terpinen-4-ol, a naturally derived monoterpenoid alcohol recognized for its biosafety and broad-spectrum antifungal activity has emerged as a promising plant-derived fungicide for controlling postharvest diseases caused by phytopathogenic fungi. The present study demonstrated that terpinen-4-ol controlled the *B. cinerea* development in friuts by inducing cell apoptosis.

The accumulation of intracellular ROS has been established as a biochemical hallmark preceding apoptotic initiation (Liang et al., 2023). Apoptosis is a classical execution pathway of cell death and a highly regulated process that occurs naturally in multicellular organisms (Tkachenko, 2024). Previous studies have demonstrated that treatment with potato glycoside alkaloids elicited significant upregulation of NADPH oxidase (NOX) and superoxide dismutase (SOD), which ultimately leads to apoptosis in *F. solani* (Sun et al., 2024). Terpinen-4-ol induces ROS accumulation in *F. sambucinum* and *F. solani*, then further activated the caspase in *Penicillium italicum*, a critical protease to initiate apoptosis program (Duru et al., 2003). The ROS-mediated apoptosis in *A. flavus* may involve mitochondrial cytochrome c translocation to the cytosol, where it initiates apoptosome assembly (Ma et al., 2022). However, direct evidence confirming apoptotic progression in these fungal species remained elusive. In the present study, *B. cinerea* emitted green fluorescence after terpinen-4-ol treatment. In contrast, only a few spores in the control emitted green fluorescence, and the fluorescence was weak and sparse. This result revealed that terpinen-4-ol elicited ROS accumulation, which further induced apoptosis. This conclusion is supported by the reduced sensitivity of the *ΔBcMca1* mutant to terpinen-4-ol, indicating that BcMca1 plays a crucial role in mediating apoptosis in response to oxidative stress. Thus, both assays above confirmed that terpinen-4-ol triggered cell apoptosis in *B. cinerea*. The results were consistent with our previous observations of perillaldehyde-mediated apoptosis (Wang et al., 2023). Therefore, we speculated that terpinen-4-ol induces apoptosis mediated by ROS accumulation in *B. cinerea* through a metacaspase-dependent pathway. This mechanism is similar to that observed in *A. flavus* and yeast, where the metacaspase Yca1 is involved in programmed cell death under oxidative stress (Lam and Sherlock, 2023; Qu et al., 2019). Our study provides further evidence of the conserved role of metacaspases in fungal apoptosis and highlights the potential of terpinen-4-ol as a natural compound for inducing apoptosis in plant pathogens.

Many natural products have been demonstrated to simultaneously trigger autophagy and apoptosis in mammalian cells, mainly through modulation of the mTOR signaling pathway (Qin et al., 2024; Zhu et al., 2022). Polyphenolic agents, including resveratrol and (−)-Epigallocatechin-3-gallate induces apoptosis and autophagy in cells by regulating Akt/mTOR signaling pathway (Yang et al., 2022; Yin et al., 2021). Triterpenoid and flavonoid derivatives, such as celastrol, apigenin and genistein induces apoptosis and autophagy via the ROS/JNK signaling pathway or endoplasmic reticulum stress (Kayacan et al., 2021; Liu et al., 2019; Wu et al., 2024). In addition to inducing apoptosis via terpinen-4-ol treatment, endoplasmic reticulum autophagy and autophagy levels were significantly elevated. We hypothesised that excess autophagy leads to apoptosis. However, our current approach to evaluate metacaspase-mediated apoptosis through radial growth inhibition assays of deletion mutants on terpinen-4-ol-containing plates has certain limitations. While this indirect method suggests that terpinen-4-ol-induced apoptosis requires metacaspase activity, it cannot provide definitive mechanistic evidence. Sousa et al. demonstrated that YCA1 deletion strains exhibit significantly increased resistant to nickel oxide nanoparticles (NiO NPs) toxicity, which suggests that NiO NPs-induced apoptosis is caspase-dependent (Sousa et al., 2019).

Fungal cell membrane, enriched with diverse lipids, plays a critical role in maintaining cellular physiology (Ren et al., 2024). We found that a significant increase in PI influx following terpinen-4-ol treatment indicated irreversible membrane damage. This result was consistent with previous publications, while terpinen-4-ol showed a stronger ability to induce cell membrane damage to spores of *B. cinerea* than in *A. flavus* (Ren et al., 2024). Due to the lipophilic nature of fungal cell membranes, it is one of the main targets of essential oils (Tian et al., 2012; Yu et al., 2015).

## Conclusion

In summary, terpinen-4-ol exhibits potent antifungal activity against *Botrytis cinerea* through multiple mechanisms, including the disruption of cell membrane integrity, induction of ROS accumulation, activation of apoptosis via the metacaspase *BcMca1* pathway, and induction of ER-phagy and non-selective autophagy. Since our study only evaluated terpinen-4-ol-induced apoptosis in metacaspase mutants, these findings have certain limitations, and further experiments are required for validation. Nevertheless, the ROS induction assay, combined with mutant sensitivity assay and annexin V-PE staining, confirmed that terpinen-4-ol triggers apoptosis through the metacaspase-dependent pathway. These findings highlight the potential of terpinen-4-ol as a natural and effective antifungal agent for controlling plant pathogens and provide a foundation for further exploration of its application in agricultural and food preservation settings.

## Data Availability

'The original contributions presented in the study are included in the article/Supplementary material, further inquiries can be directed to the corresponding authors.
