# Supplementary material for: Terpinen-4-ol triggers autophagy activation and metacaspase-dependent apoptosis against Botrytis cinerea
Source: Front Microbiol. 2025 Aug 29;16:1600831. doi: 10.3389/fmicb.2025.1600831 (PMC12425932; doi:10.3389/fmicb.2025.1600831)
Supplement: Supplementary file 1 [file Table_1.docx]

Supplementary materials

Figure legend


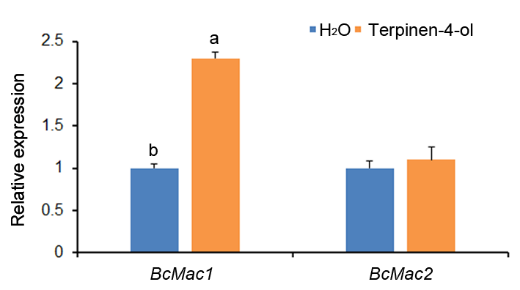


Figure S1 The relative expression levels of *BcMac1* and *BcMac2* in mycelia treated with terpinen-4-ol for 4 hours are shown. Vertical bars indicate the standard error (SE), and values labeled with different characters are significantly different (p < 0.05).


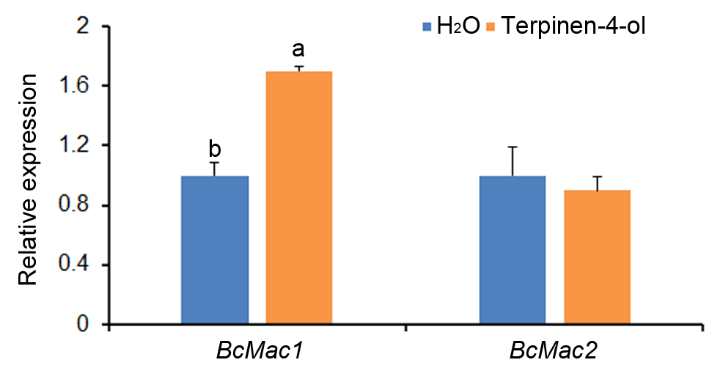


Figure S2 The relative expression levels of *BcMac1* and *BcMac2* were analyzed following 24 hours of terpinen-4-ol treatment during pathogenicity assays on tomato leaves. Vertical bars indicate the standard error (SE), and values labeled with different characters are significantly different (p < 0.05).
